# Supplementary material for: Reconciling Mining with the Conservation of Cave Biodiversity: A Quantitative Baseline to Help Establish Conservation Priorities
Source: PLoS One. 2016 Dec 20;11(12):e0168348. doi: 10.1371/journal.pone.0168348 (PMC5173368; doi:10.1371/journal.pone.0168348)
Supplement: S1 Dataset — (ZIP) [file pone.0168348.s002.zip › Taxa/Serra Norte/SN_2013/N4E/Lista N4E-38.pdf]

CAVIDADE N4E-0038

Classe	Ordem	Fam/Outros	Gên/Outros	Espécie	S-U	U-S	Zon.
Arachnida	Acari	Ixodidae		jovem	X		E
Arachnida	Amblypygi	Charinidae	*Charinus*	sp.1	1		E
Arachnida	Amblypygi	Phrynidae	*Heterophrynus*	*longicornis*		X	E
Arachnida	Araneae	Corinnidae		jovem	1	1	E
Arachnida	Araneae	Filistatidae		sp.1	X	X	E
Arachnida	Araneae	Mysmenidae	*Microdipoena*	sp.1	X		E
Arachnida	Araneae	Pholcidae	*Metagonia*	sp.	X	X	E
Arachnida	Araneae	Salticidae		sp.6	X		E
Arachnida	Araneae	Theridiosomatidae	*Plato*	sp.		X	E
Arachnida	Pseudoscorpiones	Olpiidae		sp.		X	E
Chilopoda	Scutigeromorpha	Pselliodidae	*Sphendononema*	*guildingii*		X	E
Insecta	Blattodea			jovem	1		E
Insecta	Coleoptera	Elateridae		sp.2	X		E
Insecta	Coleoptera	Staphylinidae		sp.		X	E
Insecta	Diptera	Psychodidae	Phlebotominae	sp.	X		E
Insecta	Diptera	Psychodidae	Psychodinae	sp.		X	E
Insecta	Diptera	Tethinidae		sp.		X	E
Insecta	Diptera	Tipulidae		sp.		X	E
Insecta	Heteroptera	Reduviidae	Reduviinae	sp.	1		E
Insecta	Hymenoptera	Formicidae	*Camponotus*	sp.2	X		E
Insecta	Hymenoptera	Formicidae	*Dolichoderus*	aff. *bispinosus*		X	E
Insecta	Hymenoptera	Formicidae	*Gnamptogenys*	sp.1	X		E
Insecta	Hymenoptera	Formicidae	*Pheidole*	sp.		X	E
Insecta	Isoptera	Termitidae	*Nasutitermes*	sp.	X	X	E
Insecta	Lepidoptera	Noctuoidea		sp.5	1		E
Insecta	Lepidoptera	Tineoidea		sp.1	X		E
Insecta	Neuroptera	Myrmeleontidae		jovem	1	X	E
Insecta	Orthoptera	Phalangopsidae	*Paraclodes*	jovem	1		E
Insecta	Orthoptera	Phalangopsidae	*Phalangopsis*	sp.	1	X	E
Amphibia	Anura	Strabomantidae	*Pristimantis*	cf. *fenestratus*	1		E
Amphibia	Anura			sp.		1	E
Mammalia	Chiroptera			sp.	2	1	E
